# Unlocking Vitality: A Comprehensive Review of Vitamin D’s Impact on Clinical Outcomes in Critically Ill Children

**DOI:** 10.7759/cureus.60840

**Published:** 2024-05-22

**Authors:** Keta Vagha, Amar Taksande, Sham Lohiya, Chaitanya Kumar Javvaji, Jayant D Vagha, Punam Uke

**Affiliations:** 1 Pediatrics, Jawaharlal Nehru Medical College, Datta Meghe Institute of Higher Education and Research, Wardha, IND

**Keywords:** pediatric patients, intensive care, supplementation, inflammation, immune function, clinical outcomes, critically ill children, vitamin d

## Abstract

This comprehensive review explores the multifaceted role of vitamin D (VD) in critically ill children, examining its implications for clinical outcomes. Although this substance has long been known for its function in maintaining bone health, it is now becoming more widely known for its extensive physiological effects, which include immune system and inflammation regulation. Observational research consistently associates VD levels with outcomes like duration of hospitalization, mortality, and illness severity in critically ill pediatric patients. Mechanistically, it exerts anti-inflammatory and endothelial protective effects while modulating the renin-angiotensin system. Increasing VD levels through supplementation presents promise as a therapeutic strategy; however, further research is necessary to elucidate optimal dosage regimens and safety profiles. This review emphasizes the significance of comprehending the intricate relationship between VD and critical illnesses among pediatric populations.

## Introduction and background

Pediatric critical care poses a formidable challenge within the medical landscape, where maintaining the delicate balance between life and death relies heavily on navigating intricate physiological disturbances. According to the World Health Organization (WHO), critical illness in pediatric patients encompasses a spectrum of conditions that necessitate urgent medical intervention to avert organ dysfunction or failure. In medical emergencies, conditions characterized by significant compromise in airway, breathing, circulation, or acute deterioration of consciousness are deemed critical. These conditions manifest in various forms, including but not limited to central cyanosis, apnea, hypoxemia, unconsciousness, severe respiratory distress, shock, active bleeding requiring transfusion, or seizures [1].

It has long been known that the fat-soluble steroid vitamin D (VD) plays a traditional role in maintaining calcium and phosphate homeostasis, which is essential for bone mineralization and skeletal health. The current study has revealed its broader significance in several physiological systems, especially immunological control and inflammatory modulation [2]. VD works by attaching itself to the VD receptor (VDR), which is expressed in many different types of cells, including immune cells like macrophages, T lymphocytes, and B lymphocytes [3]. When the VDR is activated, signaling events alter gene transcription and affect cytokine production, cellular differentiation, and proliferation.

Systemic inflammation and dysregulated immune responses play a crucial part in the morbidity and mortality of critically ill children. Thus, VD's immunomodulatory qualities present a promising treatment option for reducing severe inflammation and enhancing clinical results [4]. Moreover, VD deficiency is increasingly recognized as a prevalent issue among pediatric populations, particularly in critically ill children with limited sun exposure or underlying chronic conditions [5]. Suboptimal VD levels may hamper recovery because they increase infection vulnerability and affect the etiology of inflammatory illnesses.

The importance of VD supplementation in pediatric critical care is underscored by its potential to address underlying deficiencies and modulate immune function, thereby potentially improving outcomes in critically ill children [5]. However, the optimal dosing regimens, timing of initiation, and duration of therapy remain subjects of ongoing research and debate. Furthermore, individual variations in VD metabolism and responsiveness necessitate a personalized approach to supplementation tailored to each patient's specific needs and clinical context [6].

Given the complexity of pediatric critical care and the evolving understanding of VD's role in immune modulation and inflammation regulation, a comprehensive review of the existing literature is warranted. This review aims to inform clinical practice, guide nutritional management strategies, and stimulate further research endeavors to optimize outcomes for critically ill children by synthesizing available evidence and elucidating underlying mechanisms.

## Review

Metabolism of VD

**Figure 1:**
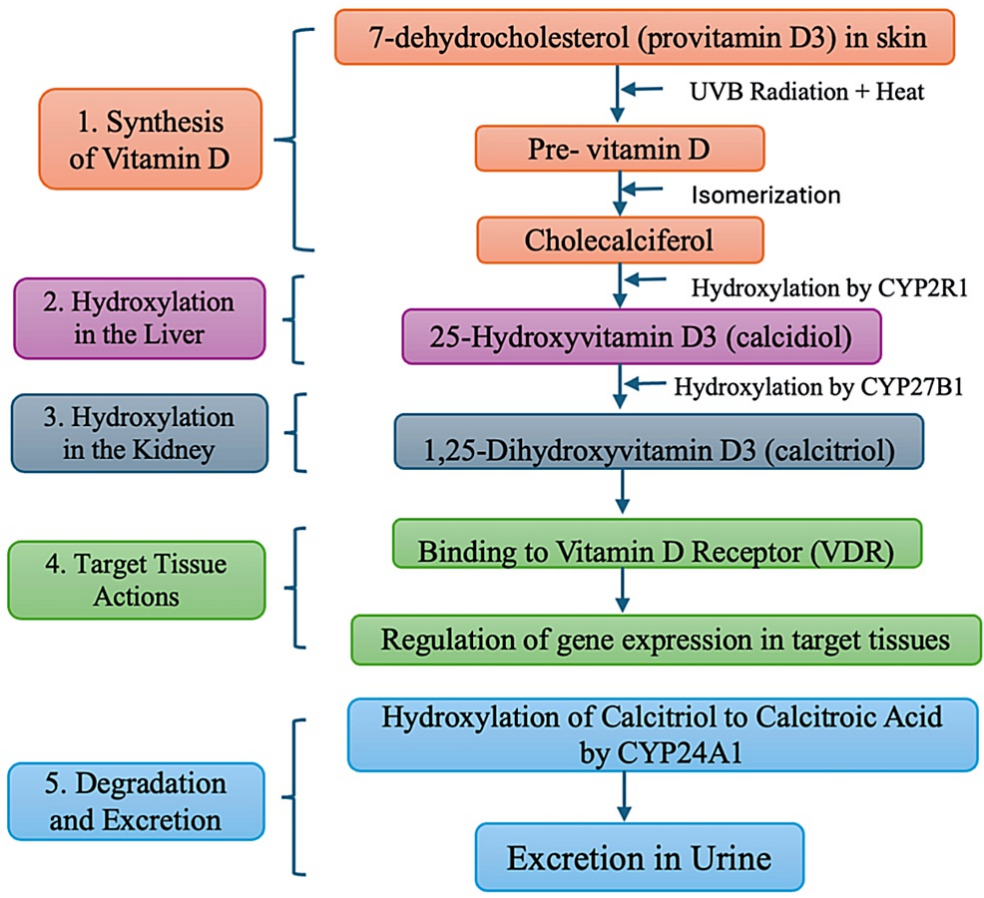
Flowchart depicting the metabolism of vitamin D Image credit: Dr. Keta Vagha UVB: ultraviolet B

Comprehending the metabolism of VD is vital for understanding its physiological functions and clinical significance. This metabolic process encompasses multiple stages, including synthesis, activation, transportation, and degradation, predominantly occurring in the skin, liver, and kidneys (Figure 1).

Synthesis of VD

VD can be synthesized in the skin upon exposure to ultraviolet B (UVB) rays or obtained from dietary sources. In the process of VD synthesis, 7-dehydrocholesterol, also known as provitamin D3, is primarily located in the epidermis and serves as the initial substrate for the cutaneous synthesis process [7]. UVB radiation facilitates the conversion of 7-dehydrocholesterol into pre-VD3, which then undergoes a heat-dependent isomerization process to form VD3 (cholecalciferol) [8]. Upon binding to VD binding protein (DBP), VD3 is released into the bloodstream and transported to the liver for further metabolic processing.

Hydroxylation in the Liver

The liver synthesizes 25-hydroxyvitamin D3 (25(OH)D3), also referred to as calcidiol, by hydroxylating VD3 at the C-25 position using the enzyme 25-hydroxylase (CYP2R1) [9]. Since 25(OH)D3 is the predominant circulating form of VD and serves as the primary indicator of VD status in clinical contexts, this hydroxylation process is crucial for storing and transporting VD.

Hydroxylation in the Kidneys

The subsequent step in VD activation occurs primarily in the kidneys through a secondary hydroxylation process. The enzyme 1α-hydroxylase (CYP27B1), located in the proximal renal tubules, facilitates the hydroxylation of 25(OH)D3 at the C-1 position. This enzymatic action results in the production of 1,25-dihydroxyvitamin D3 (1,25(OH)2D3), also known as calcitriol, which is the biologically active form of VD [10]. This conversion process is meticulously regulated by various factors, including serum calcium and phosphate levels, fibroblast growth factor 23 (FGF23), and parathyroid hormone (PTH) to uphold calcium and phosphate equilibrium.

Target Tissue Actions

Following production, 1,25(OH)2D3 binds to the VD receptor (VDR), a nuclear receptor found in several target tissues, including the kidneys, intestines, bones, and immune cells, to carry out its biological functions [11]. Through pleiotropic effects, activation of the VDR modifies the expression of genes linked to immunological response, hormone secretion, cellular proliferation and differentiation, calcium absorption, bone mineralization, and immune response. These changes in gene expression impact a variety of physiological processes.

Degradation and Excretion

The kidneys play a central role in the metabolic inactivation of 1,25(OH)2D3, the active form of VD. This process involves the enzyme 24-hydroxylase (CYP24A1), which hydroxylates 1,25(OH)2D3 at the C-24 position to produce calcitroic acid [12]. Calcitroic acid, a water-soluble metabolite, is excreted in the urine, completing the VD metabolic pathway.

Types of VD

VD plays a significant role in various physiological functions. There are various forms of VD, each with distinct roles and sources. From a medical perspective, understanding these different forms is essential for assessing VD status and managing related health conditions.

VD2 (Ergocalciferol)

When exposed to UV light, plants produce vitamin VD2. In comparison to VD3, it has lower biological activity [13]. Supplements and fortified foods containing VD2 are widely available, particularly for those who follow vegetarian or vegan diets.

VD3 (Cholecalciferol)

The most physiologically active form of VD, VD3, is mainly obtained from animal sources, including egg yolks, liver, and fatty fish. When exposed to sunlight, the skin synthesizes it [14]. When it comes to increasing serum levels of 25-hydroxyvitamin D, the main form of VD that is circulating, VD3 is superior to VD2 [15]. It is the recommended form for treating VD insufficiency and is frequently seen in supplements.

Calcidiol (25-Hydroxyvitamin D)

Calcidiol is the primary circulating form of VD and is produced in the liver through the hydroxylation of VD2 or VD3. It serves as a marker for assessing VD status in the body [2]. The measurement of serum 25-hydroxyvitamin D levels is commonly used to evaluate VD status and guide supplementation.

Calcitriol (1,25-Dihydroxyvitamin D)

The hydroxylation of calcidiol in the kidneys yields calcitriol, the active hormonal form of VD. It is essential for immune system function, bone metabolism, and calcium homeostasis [16]. In addition to promoting bone mineralization and immune response modulation, calcitriol controls the intestinal absorption of calcium and phosphate. Conditions, including secondary hyperparathyroidism and chronic renal disease, are treated using synthetic analogs of calcitriol, such as paricalcitol and calcitriol itself.

Calcifediol (25-Hydroxyvitamin D3)

The prohormone version of VD called calcifediol is being studied for possible medical applications. In the kidneys, it is similarly metabolized to calcitriol as calcidiol [17]. Studies indicate that supplementing with calcifediol may be a more effective way to raise serum levels of this vitamin than supplementing with cholecalciferol (VD3), especially in individuals with malabsorption problems or obesity [18].

Sources of VD

VD, a crucial nutrient for overall health, can be obtained from various sources, including sunlight exposure, dietary intake, and supplements. Understanding these sources is essential from a medical perspective to ensure adequate VD levels and prevent deficiency-related health issues.

Sunlight Exposure

The primary natural source of VD is sunlight. A cholesterol derivative in the skin is transformed into VD3 when exposed to UVB radiation from sunlight (wavelength: 290-315 nm) [4]. However, several variables, including the time of day, season, latitude, skin pigmentation, and exposed area, affect the body's capacity to manufacture VD from sunshine. Generally speaking, spending 10 to 30 minutes in the sun without sunscreen two to three times a week will help maintain sufficient levels of VD [19]. Older children are advised to have 30-45 minutes of sunlight exposure daily, five days a week, during the midday (11 a.m. to 3 p.m.) hours. Infants should receive 17-30 minutes of solar exposure daily [20].

Dietary Sources

While very few meals naturally contain significant levels of VD, some foods can also provide you with VD. Dietary sources of VD include, for instance: fatty fish, including tuna, mackerel, and salmon [21]; cod liver oil, which has high levels of vitamins A and D3; egg yolks, which are slightly VD-rich; and fortified foods, such as yogurt, milk, and orange juice, which contain VD2 or VD3 during processing to improve nutritional content [22].

Supplements

VD supplements are widely available and are commonly used to prevent or treat VD deficiency. These supplements typically contain either VD2 or VD3. While both forms can effectively raise serum 25-hydroxyvitamin D levels, VD3 is generally considered more effective at increasing and maintaining VD status than VD2 [14]. Supplements are available in various doses, including tablets, capsules, and liquid forms, and should be taken under medical supervision to avoid toxicity.

Fortification

A public health policy called fortification of foods with VD aims to lower the prevalence of VD insufficiency, especially in populations with low levels of sun exposure or inadequate dietary consumption. Foods, including milk, breakfast cereals, and plant-based substitutes like soy and almond milk, are frequently fortified with VD. Although fortification levels differ by nation and law, they usually seek to supply a sizable amount of the daily recommended consumption of VD [23].

VD and sunlight

The best time of day to get VD from sunlight varies depending on several factors, such as skin type, season, location, and individual health concerns. While exposure to sunlight is necessary for the skin's natural synthesis of VD, too much sun exposure can cause sunburn and raise the risk of skin cancer. Thus, balancing sun damage limitation and getting enough VD is critical.

The optimal time of day for the body to make VD from sunlight is when the sun's UVB rays are most significant, usually between 10 a.m. and 3 p.m. [4]. The season and geographic location, however, impact this timing. In areas nearer the equator, where the sun's rays are more direct all year round, shorter exposure periods might be adequate. On the other hand, longer exposure times might be required to generate enough VD during the winter and at higher latitudes [19].

It is crucial to remember that the duration of time needed in the sun varies according to skin type, age, and the amount of exposed skin. Fair-skinned people synthesize VD more quickly than people with darker skin tones, and older adults are less capable of skin synthesis than younger people [24]. Synthesizing more VD is also possible when larger areas of skin, such as the arms, legs, and torso, are exposed.

Sun protection is crucial to reducing the danger of skin damage and skin cancer, even with the possible benefits of sunshine exposure for VD synthesis. Wearing protective clothes, finding shade during peak sun hours, and using sunscreen with a sun protection factor (SPF) of 30 or higher can all help minimize UV exposure while allowing for adequate production of VD [25]. It may be challenging to obtain VD from sunlight alone for people who reside in areas with low UVB radiation or who have minimal sun exposure. Food sources and supplements can be required to maintain appropriate VD levels in such circumstances [22].

Benefits of VD

Sunlight exposure causes the skin to produce VD, also known as the "sunshine vitamin," in the form of UVB radiation. It is essential for many physiological functions in the body, and getting VD from sunshine has numerous health advantages from a medical standpoint.

Bone Health

The most well-known function of VD is that it helps maintain healthy bones. In order to maintain adequate bone mineralization and prevent diseases like osteoporosis and osteomalacia, VD helps control the absorption of calcium and phosphorus in the intestines [4].

Muscle Function

Strength and muscle function are further aspects of VD's effect. Particularly in older persons, adequate VD levels are linked to increased muscle strength and balance, which lowers the risk of falls and fractures [26].

Immune System Support

In addition to its immunomodulatory properties, VD is essential for immune system support. It improves the body's capacity to combat infections and lessen inflammation by regulating innate and adaptive immune responses [27].

Mood and Mental Health

According to certain studies, VD might have an impact on mental health and mood control. Seasonal affective disorder (SAD), depression, and other mood disorders have been linked to lower levels of VD [28]. Increased VD production from sunshine exposure may help elevate mood and lessen depressive symptoms.

Cardiovascular Health

There is evidence that VD may be beneficial for the heart. Lowered risks of stroke, hypertension, and cardiovascular disease are linked to adequate VD levels [29]. Blood pressure regulation, endothelial function enhancement, and inflammation reduction are all potential benefits of VD for cardiovascular health.

Cancer Prevention

Research has been done on VD's possible contribution to cancer prevention. Adequate levels of VD have been linked to a lower risk of prostate, breast, and colorectal cancers, among other cancers [30]. One potential strategy for preventing cancer is the regulation of cell proliferation, differentiation, and death, which may be aided by VD.

Autoimmune Disorders

In addition, VD may help to prevent and treat autoimmune diseases like multiple sclerosis, type 1 diabetes, and rheumatoid arthritis. Due to its immunomodulatory properties, VD may be able to control autoimmune reactions and lower inflammation [31].

Insulin Regulation

Insulin, a pancreatic hormone, regulates blood glucose levels by facilitating glucose uptake into cells for energy utilization or storage. Numerous investigations have explored the correlation between VD and insulin secretion, emphasizing the critical role of adequate VD levels in sustaining optimal insulin functionality. A principal mechanism by which VD impacts insulin secretion is by modulating the expression and activity of insulin-producing beta cells within the pancreas. Studies indicate the presence of VDRs in pancreatic beta cells, signifying the direct involvement of VD in regulating insulin synthesis and secretion [32]. Activating these receptors by VD triggers the expression of genes associated with insulin production, thereby augmenting insulin secretion [33].

Moreover, VD is crucial in regulating calcium homeostasis, essential for insulin secretion from pancreatic beta cells. Calcium ions act as signaling molecules that prompt the exocytosis of insulin-containing vesicles in response to elevated blood glucose levels. VD facilitates calcium influx into beta cells through various channels, promoting insulin release [34]. VD significantly influences both the sensitivity and secretion of insulin. Sufficient VD levels correlate with enhanced activity of pancreatic beta cells, leading to improved glucose metabolism and insulin production.

Conversely, insufficient levels of VD have been linked to impaired insulin secretion and an increased risk of type 2 diabetes. This association is attributed to several mechanisms, including regulating calcium homeostasis, crucial for insulin release, and the presence of VDRs on pancreatic beta cells. Ensuring adequate VD levels through supplementation or sunlight exposure may promote better insulin secretion and glucose regulation [35].

Furthermore, VD possesses anti-inflammatory properties that safeguard pancreatic beta cells from inflammation-induced damage. Chronic inflammation, implicated in insulin resistance and type 2 diabetes pathogenesis, is mitigated by VD within the pancreas, thereby preserving beta cell function and insulin secretion [36]. Observational studies have consistently shown an association between VD deficiency, impaired insulin secretion, insulin resistance, and heightened risk of type 2 diabetes [37,38]. Conversely, supplementation with VD has demonstrated improvements in insulin sensitivity and beta cell function among individuals deficient in VD [39]. 

Definition of VD deficiency

Children's VD status is categorized depending on their serum 25(OH)D levels, with <12 ng/mL indicating deficiency, 12-20 ng/mL indicating insufficiency, and >20 ng/mL indicating adequacy. In 2016-2018, a Comprehensive National Nutrition Survey (CNNS) included around 35,000 children aged 1-19 from across India [40]. VD deficiency is defined as serum 25OHD <12 ng/mL in children [35]. These findings were lower than the proportions reported in studies conducted in hospitals and communities in India. Adolescents had the most significant gender disparity, with 34% of girls and 14% of boys reporting deficiencies. 

Recommendations

According to the earlier Indian Academy of Pediatrics (IAP) Guidelines and Global Consensus Guidelines, the Group pointed out that, especially regarding skeletal outcomes, there needs to be more reliable scientific data to establish whether these serum 25(OH)D cutoff levels are suitable [41]. In most known Indian research, adverse clinical outcomes and low serum 25(OH)D levels are characterized by different cutoffs ranging from 5 to 20 ng/mL [20]. Studies have demonstrated the negative relationship between blood VD and PTH [42,43].

The CNNS survey's estimation of the burden of VD deficiency did not consider other variables like inflammation and obesity. The Group decided that, following previous guidelines and the CNNS survey cutoff, a level of less than 12 ng/mL may be used to define VD deficiency without accurate serum 25(OH)D cutoffs. It is seen desirable or sufficient to have serum 25(OH)D >20 ng/mL as a buffer during times of stress and seasonal cycles [40,41].

Estimation of VD 

Worldwide, automated immunoassay is the most widely used technique in laboratories [44]. It is reasonably priced, easy to use, and requires a small sample amount. The main disadvantage is that it uses polyclonal antibodies, which are impacted by variations in the amounts of proteins in the body. However, 25(OH)D2 and 25(OH)D3, the two forms of VD, cannot be distinguished by immunoassays. Throughout infancy, immunoassay determination of 25(OH)D levels may not be a feasible technique due to the relatively more significant amounts of 25(OH)D2 in neonates [45]. Liquid chromatographic techniques, such as liquid chromatography with ultraviolet detection (LC-UV) or liquid chromatography-tandem mass spectrometry (LC-MS/MS), can overcome these drawbacks [46].

Hypervitaminosis and VD Toxicity

Furthermore, the potential for VD intoxication persisted as a cause for concern even though the therapeutic indications for VD supplementation have grown over the past 10 years. The Group believed that in order to diagnose VD intoxication, urine excretion cutoffs should be defined in addition to serum 25(OH)D levels alone. A serum 25(OH)D threshold of more than 100 ng/mL combined with concurrent hypercalcemia and hypercalciuria is considered VD toxicity. Serum concentrations between 50 and 100 ng/mL are uncommon without supplementation and should alert the physician to cease taking additional VD [20].

Dosing of VD

There needs to be more international consensus regarding the optimal level of VD supplementation, with recommendations varying among nations, spanning from 400 to 2000 IU daily [47]. Serum 25(OH)D levels rise by 15-25 nmol/L on average over a few weeks or months after taking a generally accessible and safe dose of 25 μg VD3 (1000 IU) [48].

Monitoring blood or urine calcium levels or renal function is unnecessary if VD supplementation occurs at the above-recommended levels [49]. Similarly, there is no single, widely accepted safe level of VD supplementation. The Endocrine Society establishes a daily upper limit of 10,000 IU, despite the Institute of Medicine (IOM) and the European Food Safety Authority supporting a maximum of 4000 IU/day (100 μg) [46]. Most countries have prudently set an acceptable maximum dose for adults at 50 μg/day (2000 IU) [46]. Nevertheless, this limit was set without thorough research clarifying toxicity or dose-response connections. Remarkably, little evidence indicates that daily dosages up to 125 μg (5000 IU) have significant adverse consequences [50]. Research indicates that taking 89.3 μg (3571 IU) daily will not result in hypercalcemia or other symptoms associated with hypervitaminosis D [51]. Small studies have demonstrated that extended daily ingestion of up to 250 μg (10,000 IU) of VD does not yield harmful effects in healthy persons [48].

The guidelines provided by the Indian Academy of Pediatrics emphasize daily treatment as the primary management approach rather than boluses or weekly methods. For children under one year of age, a daily dose of 2000 IU of VD3 is recommended, while for children above one year of age suffering from nutritional rickets or symptomatic VD deficiency, a daily dose of 3000 IU is recommended. At least 12 weeks of therapy is advised, while some children may need more time to achieve normal serum 25(OH)D and alkaline phosphatase levels. Preventive VD dose recommendations for various age groups and at-risk individuals propose 50-75 mg/kg/day of calcium intake in addition to VD3 supplementation [20].

Preventive dosing of VD is crucial for various age groups and at-risk populations to support overall health and well-being. Premature neonates, requiring special care, benefit from a daily intake of 400 IU to facilitate their growth and development. Similarly, neonates aged 1 to 12 months should receive the exact dosage to promote proper bone growth and immune function. Children aged 1 to 18 years are recommended to have a daily intake of 600 IU to maintain optimal bone health and overall vitality [52].

Role of gut microbiota in enhancing VD function

VD is an essential nutrient in immune system function, bone health, calcium homeostasis, and inflammatory regulation, among other physiological functions. Although exposure to sunlight and nutrition are the main ways that VD is received, there is growing evidence that the gut microbiota may also impact the metabolism and bioavailability of this vitamin [1].

Metabolism of VD

Gut bacteria possess enzymes capable of metabolizing VD precursors into their active forms, such as calcitriol (1,25-dihydroxyvitamin D). This enzymatic activity may enhance the efficiency of VD metabolism and contribute to overall VD status in the host [53].

Modulation of Intestinal Absorption

The gut microbiota can influence the expression and activity of intestinal transporters involved in VD absorption. By promoting the expression of these transporters or modulating gut permeability, gut bacteria may increase the absorption of dietary VD and its metabolites.

Immune Regulation

VD has immunomodulatory qualities, and calcitriol, the active form of the vitamin, is essential for controlling immune responses and preserving immunological tolerance. Gut microbiota-derived metabolites, such as short-chain fatty acids (SCFAs) and bile acids, can interact with VDRs and influence immune cell function, thereby modulating the immune response to pathogens and inflammatory stimuli [54].

Role of VD in immune function

VD is well known for its complex function in immune system regulation. Extensive research has highlighted its significance in modulating adaptive and innate immune responses, thereby influencing host defense mechanisms against infections, autoimmune diseases, and inflammatory disorders. This section provides a detailed exploration of the diverse immunomodulatory functions of VD, elucidating its mechanisms of action and clinical implications.

Regulation of Innate Immunity

VD interacts with immune cells to regulate innate immune responses. When microbial pathogens or inflammatory stimuli activate macrophages, they increase the expression of the VDR and the enzyme 1α-hydroxylase (CYP27B1). This allows them to convert circulating 25(OH)D3 into 1,25(OH)2D3, the active form of VD [21]. The expression of antimicrobial peptides, which have potent antimicrobial effects against fungi, bacteria, and viruses, is increased in macrophages when the VDR is activated [55]. In addition, VD increases macrophage phagocytic activity, improves autophagy-mediated intracellular pathogen killing, and reduces the release of pro-inflammatory cytokines, all of which help to balance the clearance of infections and inflammatory responses [56].

Regulation of Adaptive Immunity

VD significantly regulates adaptive immunological responses, including T and B lymphocyte activities. T helper 1 (Th1) cells are involved in cell-mediated immunity and pro-inflammatory responses. It has been shown that VD promotes the differentiation of regulatory T (Treg) cells, which is necessary for preserving immunological tolerance and reducing inflammation while inhibiting their differentiation and proliferation [57]. Furthermore, dendritic cell maturation and function are inhibited by VD, which affects antigen presentation and T cell activation [58]. Moreover, VD has been demonstrated to reduce the generation of antibodies and the proliferation of B cells, attenuating humoral immune responses [59]. VD modulates the equilibrium between pro- and anti-inflammatory mediators, which aids in coordinating adaptive immune responses and preserving immunological homeostasis.

Clinical Implications

VD's immunomodulatory qualities have significant therapeutic ramifications for immune-mediated illnesses and infectious disorders. According to epidemiological research, there may be a link between low VD levels and a higher risk of infections, autoimmune diseases, and inflammatory conditions [27]. It has been demonstrated that taking VD supplements lowers the incidence and seriousness of respiratory tract infections, such as influenza and pneumonia, especially in people with low baseline VD levels [60]. Supplementing with VD has also been investigated as a potential adjuvant therapy for autoimmune diseases because of its anti-inflammatory and immunomodulatory qualities [61].

Why VD is called a steroid hormone?

Because of its molecular resemblance to steroid hormones like cortisol, aldosterone, and sex hormones, VD is frequently referred to as a steroid hormone. The characteristics of steroid hormones include their solubility in lipids and their capacity to bind to intracellular receptors, which controls cellular reactions and gene expression. Similarly, VD works with steroids since it binds to the intracellular VDR, a member of the superfamily of steroid hormone receptors [62]. Following its binding to the VDR, 1,25(OH)2D3, which is the active form of VD, forms a complex with the retinoid X receptor (RXR) and modulates gene transcription, resulting in subsequent biological effects [63]. Moreover, VD has a steroid-similar metabolic pathway since it is generated from cholesterol precursors and goes through enzymatic transformations to produce its active form, just like steroid hormones [64]. Consequently, the structural similarity, mode of action, and metabolic pathway of VD all support its designation as a steroid hormone; thus, VD is a misnomer. Since it can be produced internally by the skin when exposed to UV light, it is not a real vitamin. It is a steroid hormone that hydroxylases synthesize in three different sequential metabolites [65].

Association between VD status and clinical outcomes

Various research studies indicate that low levels of VD may be linked to numerous clinical outcomes, including chronic illnesses, infectious infections, and mortality. Underpinned by pertinent references, this part thoroughly investigates the relationship between VD level and clinical outcomes.

Infectious Diseases

A considerable amount of research has examined the connection between VD levels and the likelihood of contracting infectious disorders, especially respiratory tract infections. Acute respiratory diseases, such as influenza, pneumonia, and tuberculosis, are more likely to occur and worsen in those who are deficient in VD [60]. VD is a critical component in innate immune responses that mechanistically aid in pathogen clearance by stimulating macrophage activity and encouraging the synthesis of antimicrobial peptides [66]. Additionally, VD controls the synthesis of cytokines and the proliferation of T cells in the adaptive immune system, which may affect the host's capacity to mount a successful defense against pathogenic pathogens [57]. Therefore, maintaining optimal VD status may confer protective effects against various infectious diseases.

Autoimmune Diseases

VD insufficiency has been connected to several autoimmune disorders like type 1 diabetes, rheumatoid arthritis, and multiple sclerosis. By boosting immunological tolerance and inhibiting excessive inflammatory responses, VD possesses immunomodulatory qualities that may assist in lessening the progression of autoimmune diseases [67]. Additionally, VD influences T cell development, improves regulatory T cell activity, and maintains the proper ratio of pro- to anti-inflammatory cytokines, all of which work to prevent autoimmune disorders [68]. The possible therapeutic benefits of VD supplementation have been highlighted by research studies that have shown correlations between VD deficiency and increased disease activity, severity, and progression in patients with autoimmune diseases.

Chronic Conditions

The status of VD has also been associated with the onset and progression of various chronic ailments, including cardiovascular diseases, neurodegenerative diseases, and metabolic disorders. Low levels of VD have been associated with an increased risk of dyslipidemia, hypertension, type 2 diabetes mellitus, and insulin resistance, suggesting that VD may be necessary for maintaining metabolic health [36]. Moreover, VD has anti-angiogenic, anti-proliferative, and anti-inflammatory properties, potentially impeding tumor growth and metastasis in specific types of cancer [69]. Furthermore, VD exerts neuroprotective effects by promoting neuronal survival, regulating neurotransmitter synthesis, and modulating neuroinflammation, thus influencing the likelihood of neurodegenerative conditions such as Alzheimer's [70].

Impact of VD deficiency on various outcomes in critically ill children

Pediatric Intensive Care Unit (PICU) Stay

In critically unwell children, more extended hospital stays have been linked to VD insufficiency. Studies reveal that patients with low VD levels usually need to stay in the PICU longer than those with normal levels [71]. This may be explained by VD's function in immunological regulation and its possible impact on the intensity and prognosis of severe illness. Furthermore, underlying medical disorders may be made worse by VD shortage, increasing morbidity and lengthening recovery periods [72].

Duration of Mechanical Ventilation

Children who are very unwell and have low VD levels are more likely to need continuous mechanical ventilation. An insufficiency of VD has been associated with a higher occurrence and greater severity of respiratory issues [73]. Low VD levels can lead to more severe respiratory compromise in patients, requiring more extended periods of mechanical ventilation to assist breathing and enhance oxygenation [74].

Occurrence of Hospital-Acquired Infections

VD insufficiency has been linked to hospital-acquired infections in critically unwell children, such as central line-associated bloodstream infection (CLABSI) and ventilator-associated pneumonia (VAP). Immune function relies heavily on VD; its deficiencies can lead to reduced mucosal immunity and decreased synthesis of antimicrobial peptides [75]. As a result, individuals with low VD levels may be more vulnerable to illnesses contracted in the hospital due to weakened defenses against nosocomial microorganisms.

Acute Kidney Injury (AKI)

Research points to a possible connection between acute renal injury in critically unwell children and VD deficiency. Renal tissue has VDRs, and research has demonstrated that the anti-inflammatory and anti-fibrotic characteristics of VD have renoprotective benefits [16]. By encouraging inflammation, oxidative stress, and endothelial dysfunction, low VD levels may put patients at risk for renal injury and make them more vulnerable to AKI during critical illness.

Multiorgan Dysfunction

Critically unwell children may be more susceptible to multiorgan failure if they have a VD deficit. The heart, liver, and gastrointestinal tract are among the tissues and organs where VDRs are expressed, indicating a possible role for VD in preserving organ integrity and function [76]. Furthermore, in critically ill patients, VD has immunomodulatory and anti-inflammatory properties that may help reduce systemic inflammation and organ damage. Therefore, in susceptible individuals, VD deficiency may exacerbate oxidative stress, inflammation, and cellular injury, which may contribute to the etiology of multiorgan failure.

Vasoactive-Inotrope Score

The degree of hemodynamic instability and cardiovascular impairment may be reflected by more excellent maximal vasoactive-inotrope scores in critically unwell children with VD deficiency. VD's impact on cardiovascular health impacts the modulation of vascular tone, endothelial function, and myocardial contractility [77]. VD deficiency may hinder these physiological functions, increasing circulatory strain and necessitating greater dosages of vasoactive drugs to sustain hemodynamic stability during life-threatening diseases.

Mortality

Several prospective cohort studies and meta-analyses examined the relationship between VD status and mortality risk. Reduced VD levels have been repeatedly linked to higher mortality from all causes, as well as from cancer, cardiovascular disorders, and respiratory conditions [78]. Multifaceted mechanisms could be responsible for this connection, such as the pleiotropic effects of VD on immunological function, cardiovascular health, and the advancement of cancer [1]. Moreover, VD deficiency has been associated with increased rates of frailty, functional decline, and comorbidities, all of which may raise the risk of mortality for those who are affected [79]. As a result, raising VD levels may have an impact on death rates and general health outcomes.

Mechanisms underlying the impact of VD on outcomes

Complex mechanisms, involving different organ systems, cellular pathways, and molecular interactions, mediate the influence of VD on various physiological processes and clinical outcomes. With the help of pertinent references, this section offers a thorough examination of the underlying mechanisms through which VD uses to influence outcomes. These mechanisms include immunological regulation, cellular signaling, gene transcription, and tissue homeostasis.

Immune Modulation

VD is essential to control immunological responses. It affects both innate immunity and adaptive immunity. One of the main ways that VD affects results is by regulating the activity of immune cells. Immune responses can be directly modulated by VDRs, which are expressed in various immune cells [78]. Antimicrobial peptides, which have strong antibacterial qualities and support pathogen clearance, are upregulated when the VDR is activated in immune cells [79]. Additionally, VD alters the equilibrium between inflammatory and anti-inflammatory mediators by suppressing the production of pro-inflammatory cytokines while promoting the secretion of anti-inflammatory cytokines [80]. VD strengthens the host's defense mechanisms against infections, autoimmune illnesses, and inflammatory disorders by modulating immunological function and preserving immune homeostasis.

Regulation of Gene Transcription

VD has biological consequences by binding to the VDR and modifying gene transcription in target cells. Following its attachment to the VDR, 1,25(OH)2D3, the active form of VD, combines with the RXR to produce a heterodimeric complex. Subsequently, this complex binds to specific VD response elements within the target genes' promoter regions [81]. This relationship regulates gene expression, affecting several physiological functions, such as immune response, bone remodeling, calcium metabolism, and cell division [3]. Moreover, VD boosts the expression of genes associated with DNA repair, apoptosis, and antioxidant defense mechanisms. This supports cellular well-being and reduces the susceptibility to oxidative stress-induced damage [77]. VD controls many physiological pathways linked to general health and well-being by modifying gene transcription.

Regulation of Cellular Signaling

VD affects cellular signaling pathways linked to cell survival, proliferation, and differentiation. Among these pathways, the mitogen-activated protein kinase (MAPK) pathway is a significant target regulated by VD, playing a critical role in cell division, growth, and apoptosis [82]. VD inhibits MAPK signaling by preventing the phosphorylation and activation of p38 MAPK, extracellular signal-regulated kinase (ERK), and c-Jun N-terminal kinase (JNK). This inhibition slows down cell proliferation and promotes differentiation [83]. Additionally, VD influences tissue homeostasis and the development of illness by modulating the Wnt/β-catenin signaling pathway, which is involved in immunological regulation, carcinogenesis, and bone metabolism [84]. VD has pleiotropic effects on tissue homeostasis and cellular function through cellular signaling regulation, which may impact different clinical outcomes.

Maintenance of Tissue Homeostasis

VD regulates cellular differentiation, proliferation, and death, which helps to maintain tissue homeostasis. By promoting the development of several cell types, including immune cells, keratinocytes, and osteoblasts, VD improves tissue function and repair [85]. Furthermore, VD suppresses tumor growth and spread by causing apoptosis and cell cycle arrest, preventing cancer cell growth [86]. Moreover, VD controls angiogenesis, wound healing, and bone turnover, which are critical for tissue regeneration and repair [87]. Furthermore, VD promotes cellular health and vitality by improving mitochondrial function, oxidative phosphorylation, and energy metabolism [88]. VD promotes organ function and resilience by preserving tissue homeostasis, which may impact clinical outcomes in both health and disease.

Future directions

Precision Medicine Approaches

Prospective investigations should concentrate on pinpointing specific elements that impact the metabolism of VD, receptiveness to supplementation, and vulnerability to health consequences associated with VD. Precision medicine approaches, incorporating genetic, epigenetic, and environmental factors, may help personalize VD recommendations and optimize health outcomes for individuals with varying risk profiles [89].

Clinical Trials and Intervention Studies

Well-designed randomized controlled trials are essential to obtain deeper insights into the safety and effectiveness of VD supplementation across a range of clinical populations and disorders. Thorough studies are required to ascertain the most appropriate dosage schedules, how long to supplement, and the long-term effects of VD on important health outcomes like mortality, morbidity, and quality of life. Large-scale intervention studies should address these aspects comprehensively [90].

Mechanistic Studies

It is essential to conduct additional mechanistic research to understand the molecular pathways by which VD influences gene transcription, cellular signaling, immunological function, and tissue homeostasis. Knowing how VD interacts with different biological pathways may help identify new therapeutic targets and guide the creation of focused therapies for preventing and treating illnesses linked to VD deficiency [69].

Public Health Initiatives

Public health initiatives are essential to raise awareness about VD's importance and promote strategies for maintaining optimal levels. Campaigns for public education, legislators, and healthcare professionals can aid in disseminating evidence-based advice and promoting behavioral change. Collaborative efforts between healthcare providers, government agencies, and community organizations are needed to address VD deficiency as a public health priority [46].

## Conclusions

In conclusion, VD's importance in health and sickness is highlighted by the complex link between it and clinical outcomes. VD influences many physiological processes, including immune modulatory responses, gene transcription regulation, cellular signaling modulation, and tissue homeostasis maintenance. These effects affect autoimmune disorders, chronic conditions, mortality, and infectious diseases. It may be possible to improve general health outcomes by identifying the clinical consequences of VD insufficiency and optimizing VD status through preventative measures and focused therapies. Future research should concentrate on precision medicine approaches, clinical trials, mechanistic studies, and public health initiatives to further our understanding of VD physiology and apply this knowledge to effective disease prevention and management measures.
